# Improvement in insulin injection timing and glucometrics using a connected insulin cap: the *Insulclock v2.0^®^* prospective study

**DOI:** 10.3389/fendo.2026.1883791

**Published:** 2026-07-15

**Authors:** Fernando Gomez-Peralta, José Miguel Borrachero, Estefanía Santos, Cristina Abreu, Xoán Valledor, Luis Ruiz-Valdepeñas, Rosa Corcoy

**Affiliations:** 1 Endocrinology and Nutrition Unit, Hospital General de Segovia, Segovia, Spain; 2 Centro de Salud del Peral, Cartagena, Spain; 3 Endocrinology and Nutrition Service, Complejo Hospitalario Universitario de Burgos (HUBU), Burgos, Spain; 4 Research and Development Unit, Insulcloud S.L., Madrid, Spain; 5 Institut de Recerca, Hospital de la Santa Creu i Sant Pau, Barcelona, Spain; 6 Departament de Medicina, Universitat Autònoma de Barcelona, Bellaterra, Barcelona, Spain; 7 Centro de Investigación Biomédica en Red de Bioingeniería, Biomateriales y Nanomedicina (CIBER-BBN), Madrid, Spain

**Keywords:** Connected insulin pen cap, Insulin adherence, Insulin injection timing, Continuous glucose monitoring, Glycemic control

## Abstract

**Objectives:**

Matching insulin injection timing with meals to optimize postprandial glucose excursions is a daily challenge for individuals with diabetes on a multiple daily injection (MDI) regimen. We aimed to analyze the impact of using a connected insulin pen cap (CIPC) on insulin injection timing and glycemic control.

**Research design and methods:**

Pragmatic, real-life, multicenter, prospective, open-label, observational study, including one week of run-in and a 6-week follow-up, split into a two-week masked mode phase and a four-week active phase. Continuous glucose monitoring (CGM) and automatically tracked insulin injection data in individuals with insulin-treated diabetes (ITD) who started using the CIPC Insulclock. The baseline and five hours of paired CGM and rapid-acting insulin data collected from *Insulclock v2.0^®^* users were analyzed using the ROC detection methodology to identify meal events and the timing of insulin doses.

**Results:**

Of 82 recruited patients, 52 completed the study (54.4 y, 56.6% women, 60.4% with type 1 diabetes [T1D]) across three hospitals and one primary care center in Spain. The CGM glucometrics comparison between the consecutive masked and active phases showed: Glucose Management Indicator (GMI) 8.1 + 1.7 vs 7.8 + 1.4% (-0.3%, p 0.034); Time in Range 70-180 (TIR): 56.9 + 24.5 vs 61.9 + 21.6% (+5.0%, p 0.0054); Time below range <70 (TBR70): 2.5 + 3.3 vs 1.76 + 2.6% (-0.74%, p 0.0015); Time above range >180 (TAR180): 40.9 + 25.3 vs 36.6 + 22.4% (-5.3%, p 0.016). The on-time insulin injections increased: 45.5 + 15.52 to 54.4 + 16.6% (p 0.0017). The timing of insulin injection relative to the post-meal glycemic excursions shifted from +5.6 min (IQR –19.8 to +34.0) in the masked phase (n = 231 events) to –5.9 min (IQR –27.6 to +33.9) in the active phase (n = 467 events) (p = 0.023). An earlier injection was associated with a reduction in TAR180 (p = 0.042). Questionnaires measuring patients’ reported outcomes (PROs) indicated a reduction in perceived treatment burden with the use of the *Insulclock v2.0^®^* CIPC.

**Conclusions:**

The use of *Insulclock v2.0^®^* connected insulin pen cap is associated with improved insulin injection timing and glucometrics.

## Highlights

- Why did we undertake this study?
  Glycemic control in people with diabetes on multiple daily insulin injections (MDI) is often inadequate.
- What is the specific question(s) we wanted to answer?
  This study aimed to evaluate the impact of a connected insulin pen cap (CIPC) on pre-meal insulin administration timing and glycemic management.
- What did we find?
  CIPC enhanced glycemic control in T1D and T2D patients on MDI by boosting insulin adherence and promoting timely pre-meal rapid insulin use.
- What are the implications of our findings?
  CIPC systems are technological tools that can optimize self-management among persons with diabetes using MDI.

## Introduction

All individuals diagnosed with type 1 diabetes mellitus (T1D) will require lifelong daily insulin therapy, while more than a quarter of those with type 2 diabetes mellitus (T2D) also require regular insulin treatment at some stage of the disease (1). Currently, over half of individuals with T1D and nearly all with T2D who require prandial insulin use multiple daily insulin injection (MDI) regimens (1, 2). Glycemic management in this population remains predominantly suboptimal (3, 4).

Inadequate diabetes management is associated with an increased risk of long-term complications, as well as diminished quality of life and higher mortality rates (5, 6). The T1D Exchange registry showed that, even as the cohort experienced a significant improvement in HbA1c from 2016 to 2022, people with T1D maintained suboptimal HbA1c levels (2). The mean HbA1c level was 8.4% across the entire cohort and 8.6% among adolescents, suggesting that treatment adherence and individual factors may play a significant role in glycemic outcomes.

Socioeconomic variables, the complexity of treatment regimens, and concerns regarding hypoglycemia influence adherence to insulin therapy (7, 8). Errors in pre-meal insulin administration, such as missed or delayed bolus doses, pose significant challenges to achieving optimal glycemic control (9). Suboptimal adherence to insulin therapy may negatively impact quality of life and is associated with higher rates of morbidity, mortality, and increased hospitalizations resulting from acute complications (10).

Clinical guidelines recommend administering rapid-acting insulin before meals (11). Manufacturers advise administering human regular insulin 30 to 45 minutes before eating, rapid insulin analogs (RI) should be injected 15 minutes prior to a meal, while second-generation “ultrarapid” insulins (URI) are recommended for administration at the beginning of a meal or within 20 minutes thereafter (11–13).

Delayed bolus administration after the meal and excessive corrective measures can result in challenging initial hyperglycemia followed by late postprandial hypoglycemia, both of which adversely affect the physical and psychological well-being of individuals with diabetes (14, 15). Second-generation rapid-acting (“ultrarapid”) insulins can enhance postprandial glycemic control even when administered after meal initiation, thereby reducing the risk of both immediate hyperglycemia and late postprandial hypoglycemia (16–19).

Connected insulin pens and caps (CIPC), when integrated with continuous glucose monitoring (CGM) sensors, represent advancements in digital devices with the potential to improve diabetes management (20). They monitor the dosage and timing of insulin administration and can set reminders to help prevent missed or delayed injections. Both users and healthcare professionals can access comprehensive data showing the correlation between insulin delivery schedules and postprandial glucose levels (21). Scientific societies consider that CIPC can be an interesting option to improve adherence and glycemic control in the T1D population on MDI who are rejecting or before using the current standard of care, automated insulin delivery systems (AID) (20).

*Insulclock^®^* is an advanced CIPC designed to document the date, time, duration, and dosage of each injection, ensuring precise and effective insulin administration (21). Multiple studies have demonstrated that the use of the *Insulclock^®^* system is associated with improvements in glycemic control and variability, insulin therapy adherence, and quality of life among individuals with T1D and T2D who experience suboptimal glycemic control (22–24). There are no head-to-head trials comparing *Insulclock^®^* with other CIPC systems. However, key differentiators include compatibility with any disposable pen, interoperability with CGM and self-monitoring blood glucose systems, and temperature monitoring. *Insulclock^®^ 2.0* is a smaller, redesigned model featuring an intuitive, user-friendly hardware-and-software interface, enhanced accuracy, and extended battery life.

The objective of this study was to assess the effects of the updated *Insulclock^®^ 2.0* CIPC on the timing of insulin administration and glycemic management.

## Research design and methods

### Design

Pragmatic, real-life, multicenter, prospective, open-label, observational study, including one week of run-in and a 6-week follow-up, split into a two-week masked mode phase and a four-week active phase. CGM and automated insulin administration data were collected from individuals with insulin-treated diabetes after initiation of the CIPC using *Insulclock v2.0^®^* (*Insulcloud S.L.; Madrid, Spain*). Prior to the commencement of *Insulclock v2.0^®^* use, written informed consent was obtained from each participant, authorizing *Insulcloud S.L.* to collect their data and to use the anonymized, tabulated information for scientific research purposes. The study was conducted in accordance with the ethical principles of the Declaration of Helsinki. Before any study-related activities, the Research Ethics Committee of Hospital General de Segovia, Segovia, Spain, approved the study (act 10/2022).

### Population

Participants eligible for inclusion in the study must meet all of the following criteria: age between 18 and 65 years of age; have a diagnosis of diabetes mellitus managed with insulin therapy for at least one year; were using CGM as their usual diabetes care, demonstrate the ability to operate the medical device and complete all required questionnaires; have both the capacity and willingness to comply with the study protocol; and provide signed informed consent. The exclusion criteria were as follows: current or recent history of drug addiction or alcohol abuse; acute infection; or any illness or condition that, in the investigator’s judgment, might interfere with the study conduct (e.g., planned surgical procedures, steroid treatment).

### Outcomes

This study was conducted to assess the efficacy of the *Insulclock v2.0^®^* device and application in improving treatment adherence, glycemic control, and overall quality of life. The primary objective was to evaluate differences between the masked mode phase and the active mode phase with respect to the timing, dosage, and adherence to insulin administration. The Glucose Rate Increase Detector (GRID) algorithm was used to automatically detect meal glucose excursions through the rate of change (ROC) of glucose from FGM data (25). Adherence was measured by the number of ‘on-time’, delayed (‘mistimed’), and omitted (‘missed’) insulin injections per month and the percentage over the total number of injections recorded. An ‘on-time’ insulin injection was considered when a bolus insulin injection was detected within 45 min before the glycemic meal excursion. Meal-time insulin doses not detected by the glucose ROC methodology were categorized as on-time. A bolus was deemed ‘mistimed’ when the insulin injection occurred within 60 min after the meal glycemic excursion start, whereas a bolus was considered ‘missed’ when the meal excursion was not associated with an insulin injection (45 min before to 60 min after the FGM rise).

The insulin dose and the timing of administration relative to meal intake detected by CGM-derived postprandial hyperglycemic excursions were examined. The baseline and five hours of paired CGM and rapid-acting insulin data were collected from the database uploaded by the *Insulclock^®^* system. The Glucose Rate Increase Detector (GRID) algorithm was used to automatically detect meal glucose excursions using the ROC of CGM glucose data (25). Those excursions that started with a glucose level < 70 mg/dL (3.9 mmol/L) or > 250 mg/dL (13.9 mmol/L), and those without 5-hour data after meal initiation, were excluded. Insulin dose and injection timing data were further evaluated based on the type of insulin administered: rapid-acting analogs (*lispro, Humalog^®^, Eli Lilly, USA*) (RI) or ultrarapid analog (*Fiasp^®^, NovoNordisk, Denmark*) (URI).

Glycemic control was evaluated according to the international CGM consensus metrics for clinical trials defined by Battelino et al. (26), including: Glucose Management Indicator (GMI), time in range 70–180 mg/dL (3.9–10.0 mmol/L) (TIR), time above range > 180 mg/dL (10.0 mmol/L) (TAR), time below range < 70 mg/dL (3.9 mmol/L) (TBR70), standard deviation (SD) and coefficient of variation (CV) of glucose. Additionally, variables describing glycemic variability were analyzed: mean amplitude of glycemic excursions (MAGE), low blood glucose index (LBGI), and high blood glucose index (HBGI) (27).

The Insulin Treatment Satisfaction Questionnaire (ITSQ) is designed to assess satisfaction with insulin therapy through 22 items distributed across five subdomains: satisfaction with the insulin delivery device, glycemic control, hypoglycemic control, regimen inconvenience, and lifestyle flexibility. Each item is rated on a scale from 1 (extremely satisfied) to 7 (extremely dissatisfied) (28). Additionally, a questionnaire measuring patient-reported satisfaction with diabetes treatment regimens (PRSD) was used. It consisted of eight items: six measure treatment satisfaction, and two assess the perceived frequency of hyperglycemia and hypoglycemia.

A purpose-built electronic CRD was implemented for this study.

### Devices

*Insulclock^®^* is a cap attachment for insulin pens designed to accurately record both the dose and timing of insulin administration (21). The device additionally monitors insulin temperature fluctuations, records the duration of each injection, and identifies the specific pen utilized. It synchronizes seamlessly with the application, enabling automatic data logging. The application includes a reminder function to facilitate timely insulin dosing and provides personalized feedback for type 1 diabetes management and education. The *Insulclock^®^* app supports both Apple and Android operating systems. *Insulclock^®^ 2.0* is the latest version, featuring a redesigned interface, enhanced user intuitiveness, an extended battery life, and improved accuracy. In this study, participants self-administered their previously prescribed insulin using pens integrated with *Insulclock^®^ 2.0*, in accordance with standard clinical practice.

During the masked mode phase, participants were not granted access to the application, nor did they receive any notifications. During the active mode phase, participants used the app to receive alerts about potential missed insulin doses, notifications to prevent insulin stacking if a previous injection occurred within two hours, and guidance on proper injection techniques, including prompts for injections lasting less than six seconds.

The *Freestyle Libre 2^®^* (*Abbott Diabetes Care, Alameda, Ca, USA*) continuously measures interstitial glucose at one-minute intervals, with data recorded every 15 minutes. Each sensor remains effective for up to 14 days. Participants applied the FGM sensor to the upper arm.

The *Dexcom OnePlus^®^* (*Dexcom, Inc., San Diego, CA,USA*) continuously measures interstitial glucose at five-minute intervals. Each sensor remains effective for up to 10 days. Participants applied the FGM sensor to the upper arm.

### Study visits

#### Visit 1, pre-screening (week -1)

One week prior to the start of the study, all prospective study participants are screened for eligibility after signing the informed consent form (ICF) and are assigned a subject identification number. During this visit, medical history, physical examination, height, weight, vital signs, pre-existing conditions, and concomitant medications are recorded. Laboratory evaluations (serum chemistry: HbA1c, fasting glucose, lipids, kidney function) must be available within the last 2 months. Subjects who do not meet all inclusion criteria at Visit 1 are considered screening failures and are excluded from the study.

#### Visit 2, start of masked mode (week 0)

Subjects were provided with comprehensive instructions for installing and using both the medical device and the CIPC app in masked mode. During this phase, the app provided no information to participants; only access to the study procedures was available. Insulin therapy was administered as previously prescribed. Participants completed the PRSD along with the ITSQ. Assistance was given to link the Insulclock medical device with the mobile application.

#### Visit 3, start active mode (week 2)

Participants received comprehensive guidance on operating in active mode. They were encouraged to use all available system features, including alarm notifications and messaging functions for both caregivers and the research team.

#### Visit 4, final visit (week 6)

The mobile application diaries were systematically reviewed. Patients were provided with questionnaires—PRSD and ITSQ—to assess quality of life and treatment satisfaction.

Safety events were documented during each visit.

### Statistical analyses

Statistical analyses were performed using the SPSS software, version 25.0 (Chicago, IL). The level of statistical significance was set at *p* < 0.05. Continuous variables were described by the mean and standard deviation (SD), when normally distributed, or by the median, interquartile range (IQR), when not normally distributed. Categorical variables were described by the number of valid cases and percentages. Comparisons of proportions and/or frequency distributions were performed with the Chi-square test, Kruskal Wallis or the ANOVA test, as appropriate, with the *post-hoc* Bonferroni correction. To examine the influence of adherence variables (insulin injection timing) on CGM-derived metrics, linear and logistic regression were used.

### Sample size estimation

The sample size estimation was based on data from our previous multicenter study on injection adherence, which provided the variability of the proportion of on-time injections (standard deviation: s = 0.08). The protocol specified a target precision equivalent to 30% of this variability. Interpreting this requirement as 30% of the standard deviation, the corresponding margin of error was set to:

W/2 = 0.3·s=0.024

Using a two-sided alpha of 0.05 and the standard formula for estimating a mean with predefined precision:

n = ((Z·s)/(W/2))^2^

where Z = 1.96 for alpha = 0.05.

By substituting the values:

n = ((1.96·0.08)/0.024)^2^ ≈ 43 participants

Considering an expected drop-out rate of approximately 30%, we propose recruiting a total of 80 subjects for the study.

## Results

### Population

From 13th June 2023 to 19th June 2024, 82 T1D and T2D participants who had started using the CIPC were recruited (**Table 1**).

**Table 1:** Demographic and clinical characteristics and glucometrics data of study participants.

Variable	All (n = 52, 100%)	Type 1 diabetes (n = 32, 61%)	Type 2 diabetes (n = 20, 39%)	Rapid insulin analogs (n = 31, 59.6%)	Second generation `ultrarapid´ analog (n = 16, 30.7%)
Age (years), mean ± SD	54.4 ± 15.7	47.87 ± 15.4	64.26 ± 12.4	53.24 ± 16.4	44.5 ± 15.8
Sex (male), n (%)	30 (57%)	17 (53.1%)	13 (65%)	16 (51.6%)	9 (56.2%)
Duration of diabetes (years), mean ± SD	32.1 ± 16.1	34.8 ± 17.9	28.1 ± 14.3	36.8 ± 16.9	32.4 ± 17.3
Weight (kg), mean ± SD	78.5 ± 19.2	74.84 ± 16.9	84.36 ± 20.4	77.65 ± 20.3	82.7 ± 13.4
Height (cm), mean ± SD	163.6 ± 9.5	164.36 ± 8.4	162.34 ± 11.4	161.7 ± 10.5	166.5 ± 4.8
GMI (%)	7.9 ± 1.6	7.8 ± 1.7	8.0 ± 0.8	7.5 ± 1.3	8.2 ± 1.9
TIR (%)	56.8 ± 14.3	56.7 ± 25.2	57.1 ± 23.2	59.0 ± 24.5	50.0 ± 26.5
TBR70 (%)	2.4 ± 3.7	3.2 ± 4.0	1.2 ± 0.6	1.8 ± 2.4	4.8 ± 6.9
TAR180 (%)	37.1 ± 23.5	41.3 ± 27.7	30.4 ± 23.1	40.2 ± 25.8	45.1 ± 32.0
CV (%)	33.3 ± 7.5	34.5 ± 7.5	31.3 ± 5.5	32.6 ± 8.0	33.7 ± 4.5
Dexcom OnePlus® Users (n)	4	4	0	4	0

CV, coefficient of variation; GMI, glucose management indicator; TAR, time above range; TBR, time below range; TIR, time in range.

**Supplementary Figure 1** presents the study flowchart, including the causes of case loss from recruitment (n = 82) to the population that completed all follow-up and had comprehensive, analyzable data (n = 52).

**Table 1** presents the demographic and clinical characteristics of the final study population.

### Insulin therapy adherence trend: delayed and missed doses

During the masked phase, the average number of daily insulin injections was 2.5 ± 1.6, and 2.8 ± 1.8 in the active phase (T-Statistic: -1.61; P-Value: 0.114). The proportion of insulin doses administered on time improved from 45.5 ± 15.5% in the masked phase to 54.4 ± 16.6% during the active phase (T-Statistic: -3.39; p-value: 0.0017). Furthermore, there was a notable decrease in the proportions of both mistimed doses (12.7 ± 10.4% to 8.8 ± 7.9%; T-Statistic: 2.5; p-value: 0.0167) and missed doses (41.6 ± 15.2% to 35.5 ± 16.3%; T-Statistic: 3.54; p-value: 0.0011).

**Figure 1:**
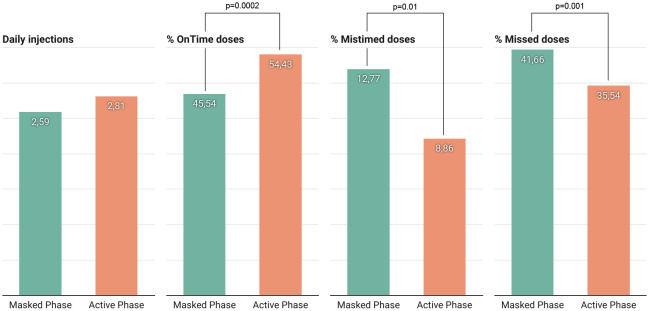
Variations in insulin adherence observed over the course of the study.

**Figure 1** describes the evolution of insulin therapy adherence data.

### Glycemic control

**Figure 2:**
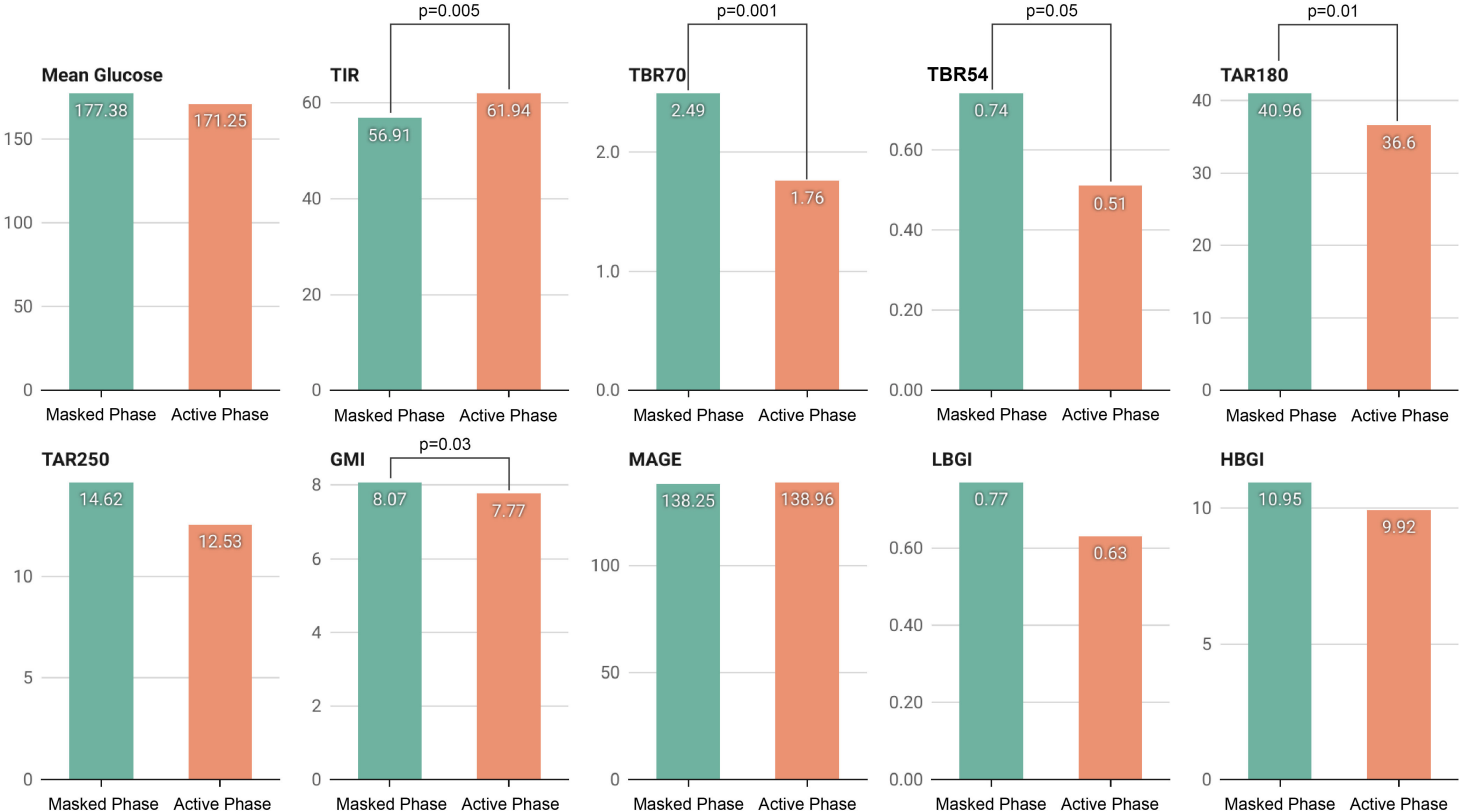
Bar charts illustrating glucometric data during both the masked phase and the subsequent active phase of connected insulin cap use.

**Figure 2** describes the evolution of CGM-derived glucometrics.

TIR increased from 56.9 ± 24.5 to 61.9 ± 21.6% (an increase of 5.0%, T-statistic: -2.94, p-value: 0.0054) when comparing the masked phase to the final active phase. GMI was reduced from 8.07 ± 1.67 to 7.77 ± 1.4% (-0.3%; T-Statistic: 2.2; p-value: 0.034). Mean glucose levels in the masked phase were 177.3 ± 50.3 and 171.2 ± 41.5 mg/dL in the final active phase (T-Statistic: 1.66; P-Value: 0.103).

Hyperglycemic exposure was also reduced. TAR180 decreased from 40.9 ± 25.3% to 36.6 ± 22.3% (t = 2.50, p = 0.0169), while TAR250 decreased from 14.6 ± 19.2% to 12.5 ± 17.0%, although this change did not reach statistical significance (t ≈ = 1.6, p ≈ 0.11).

Regarding hypoglycemia, time below range <70 mg/dL (TBR70) was significantly reduced from 2.5 ± 3.3% to 1.76 ± 2.6% (−0.74%, p = 0.0015). Similarly, time below range <54 mg/dL (TBR54) decreased from 0.74 ± 1.69% in the masked phase to 0.51 ± 1.42% in the final active phase, with a trend toward significance (t ≈ 1.9, p ≈ 0.06).

The glycemic variability index, as measured by the CV, improved from the masked phase (34.1 ± 10.2) to the active phase (31.2 ± 9.8), (T-statistic: 2.14; p-value: 0.036). MAGE, LBGI, and HBGI parameters changes did not reach statistical significance: 138.2 ± 48.3 vs 138.9 ± 44.9, p = 0.835; 0.77 ± 0.8 vs 0.63 ± 0.7, p = 0.064; 10.9 ± 9.2 vs 9.9 ± 7.8, p = 0.195, respectively.

### Dose of insulin and timing of administration in relation to meal intake

The GRID methodology identified 231 meal excursions during the masked phase and 467 during the active phase among the 47 subjects who used RI boluses.

**Figure 3:**
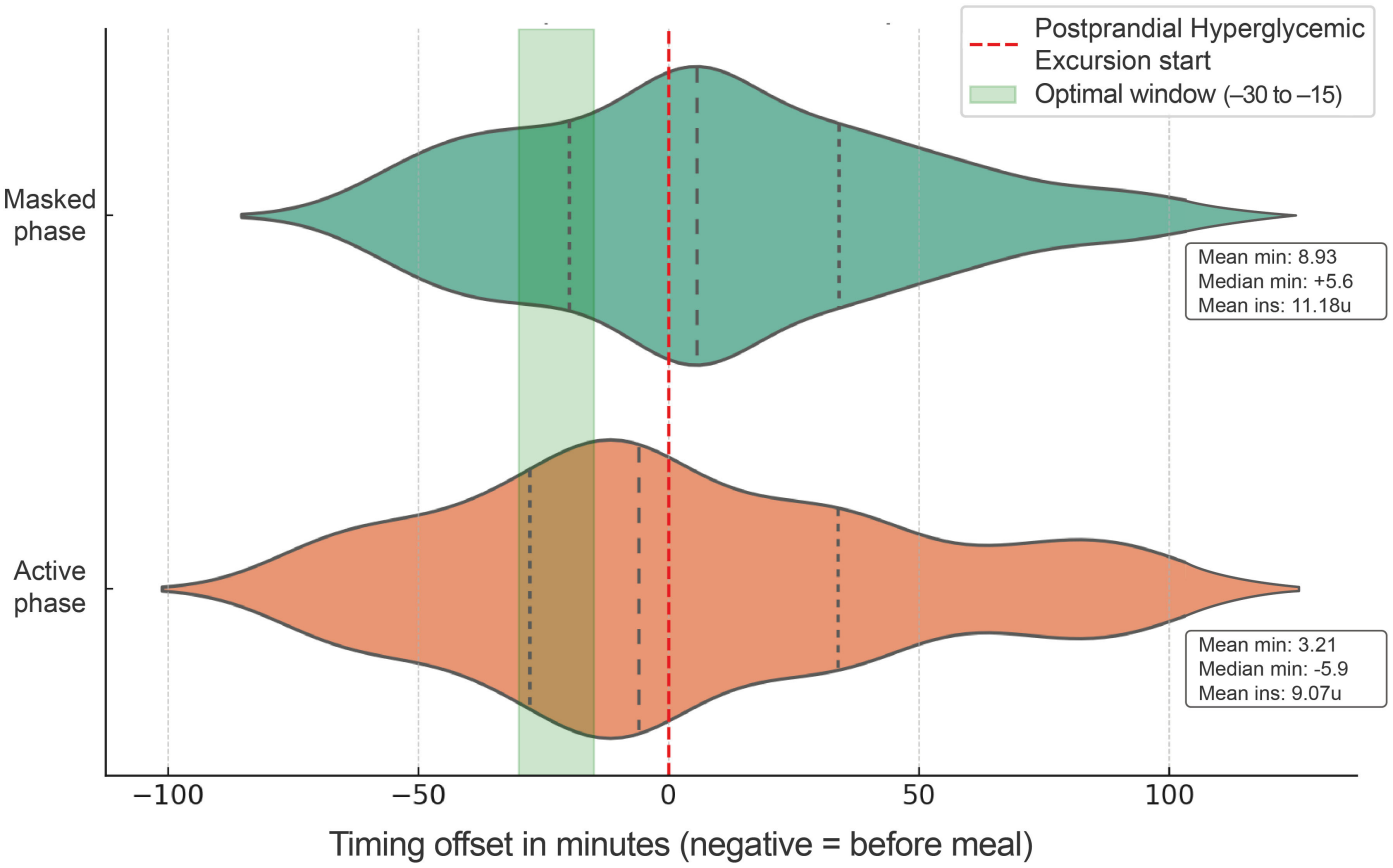
Violin plots illustrating the distributions of insulin injection timing among subjects, by study phase.

Although overall glycemic control improved, the RI dose decreased from a mean of 11.2 units during the masked phase to 9.1 units per bolus during the active phase (p = 0.002) (**Supplementary Figure 2**; **Figure 3**). Consistently, the median RI dose was reduced from 8.0 units (IQR 4.0–14.0) in the masked phase to 6.0 units per bolus (IQR 4.0–10.0) in the active phase.

Participants using the CIPC system during the active phase also advanced the timing of RI injection (**Figure 3**). During the masked phase, RI was injected at a median of 5.6 minutes after the onset of postprandial glycemic excursions (IQR –14.8 to +24.0 min). In contrast, during the final weeks of the active phase, injections occurred a median of 5.9 minutes before postprandial excursions (IQR –17.6 to +23.9 min) (p = 0.023).

The regression model analysis confirmed that an advance in injection timing was associated with improved postprandial glycemic control: TAR180 (β ≈ +0.23%/minute, p = 0.029, R² = 0.10), indicating that a 10-minute advance in RI injection was associated with a 2.3% reduction in TAR180. A trend for TIR improvement was also found (β ≈ −0.19% TIR/minute, p = 0.059, R² = 0.064) (**Supplementary Figure 2**).

The analysis of insulin dose and injection timing data based on the type of insulin administered showed that RI users (meal events n= 559) changed the insulin dose and injection time from 7 units +5.1 minutes after the postprandial hyperglycemic excursion start to 6 units administered –9,3 minutes before (p-value for insulin dose difference 0.068; p-value for insulin timing 0.050). The URI users (meal events n= 140) changed the insulin dose and injection time from 8,5 units, +23,4 minutes after the postprandial hyperglycemic excursion start to 5 U (p-value 0.068) administered +12,6 minutes after (p-value for insulin dose difference 0.010; p-value for insulin timing p = 0.40) (**Supplementary Figure 3**).

### Quality of life

Most ITSQ items improved from the masked phase to the active phase (**Supplementary Figure 4**). In particular, participants reported greater satisfaction with meal planning, prevention of symptomatic hypoglycemia, glucose stability, glycemic control, and ease of insulin dose selection during the active phase.

The PRSD item assessing the risk of hyperglycemic excursions indicated higher satisfaction with the CICP system during the active phase (**Supplementary Figure 5**).

## Conclusions

Adjustment of prandial insulin dose and timing based on meal composition is a central responsibility and challenge in the daily management of individuals with diabetes (29). Assessment and counseling in this context continue to pose significant challenges for healthcare professionals. The adoption of CGM technology has enabled a more detailed understanding of postprandial glucose trends. However, practical analysis in real-world settings remains complex for individuals with diabetes and their caregivers due to the need for precise documentation regarding insulin dosage and timing. The advent of coordinated integration between CGM data and CIPC now facilitates more accurate data correlation. A recent systematic review indicates that available studies consistently demonstrate potential improvements in glycemic control, high patient satisfaction, and promising cost-effectiveness (30).

Nevertheless, the behavioral and therapeutic modifications underlying the metabolic benefits associated with CIPC use remain insufficiently investigated and documented. Our prior randomized controlled trial involving individuals with T1D was the first to show that optimizing both the frequency and the timely administration of insulin injections improved glycemic control, as evidenced by a 4.6% increase in TIR (23). Several recent retrospective observational studies have confirmed an association between increased adherence to insulin therapy—as indicated by the number of insulin boluses administered—and improvements in metabolic outcomes (9, 31). The present prospective study confirmed an optimized glycemic control (TIR + 5.0%; GMI -0.3%, TBR70 -0.74%; TAR180 -5.3%; CV -2.9%), besides increased on-time insulin injections (+16.6%) and a decrease in mistimed (-3.9%) and missed doses (-6.1%).

However, the evaluation provides a comprehensive analysis of insulin injection techniques, including variations in automatically recorded insulin doses and in the intervals between injections and meals, as evidenced by postprandial hyperglycemic excursions. Several valuable insights emerge from this assessment. The improvement in metabolic control was achieved despite a reduction in the RI dose (from 11.2 to 9.1 units per bolus). Clinical experience shows that better management of insulin therapy, including matching the dose and injection time to meal amount and composition, is critical to maintaining stable blood glucose levels. Previous research shows that optimizing insulin therapy can improve glycemic control while reducing the total insulin dose (31). Minimizing exogenous hyperinsulinism in individuals managed with complex insulin regimens is an important objective to mitigate weight gain and other adverse effects (32). Regarding insulin administration timing, the current analysis indicated that rapid-acting insulin injections were administered significantly earlier, on average by 11.5 minutes, over the six-week study period. Scientific evidence shows advancing prandial insulin is clinically beneficial. Mistiming of insulin has been reported in up to 45% of RI users, and this was associated with higher rates of hypoglycemia and worse glycemic control (10). A systematic review of 13 studies from 2002 to 2022 found that preprandial insulin administration improves postprandial glycemic control and HbA1c in youth with type 1 diabetes, without increasing the incidence of hypoglycemia (33). The findings of our study demonstrate a measurable behavioral change over a brief period, attributable to the implementation of CICP technology.

Finally, the questionnaires utilized in the study indicated a decrease in the perceived burden of insulin treatment and enhanced efficacy among participants using the CIPC system.

Some study limitations must be stated. The lack of a control arm reduces the overall methodological rigor. The limited follow-up duration is also a notable limitation. With respect to the clinical application of insulin timing as outlined in the study, it is important to recognize that CGM assesses glucose variations in the interstitial fluid, rather than precisely identifying the initiation of meal consumption. A physiological delay occurs between eating and the rise in blood glucose. Additionally, there is a recognized lag between changes in blood glucose levels and those seen in the interstitial fluid (34). Several prior studies have reported that both lags average approximately ten minutes each (35). Subsequently, all intervals measured between insulin administration and the onset of postprandial glucose excursion should be extended by approximately 10 to 20 minutes when meal initiation is considered.

In summary, this pragmatic prospective study demonstrated that implementing a CIPC system can modify insulin administration management, leading to improved glycemic control within a short period. CIPC technology may be increasingly integrated into routine clinical care for individuals with diabetes who require insulin and must navigate complex treatment regimens. Further research is warranted to determine the most effective strategies for using these technologies.

## Data Availability

The raw data supporting the conclusions of this article will be made available by the authors, without undue reservation.
