# Supplementary material for: Improvement in insulin injection timing and glucometrics using a connected insulin cap: the Insulclock v2.0® prospective study
Source: Front Endocrinol (Lausanne). 2026 Jul 15;17:1883791. doi: 10.3389/fendo.2026.1883791 (PMC13414909; doi:10.3389/fendo.2026.1883791)

Supplementary files for

Improvement in insulin injection timing and glucometrics using a connected insulin cap: The *Insulclock v2.0*® prospective study.

<u>Supplementary Figure 1 presents the study flowchart, including the causes of case loss from recruitment (n = 82) to the population that completed all follow-up and had comprehensive, analyzable data (n = 52).</u>

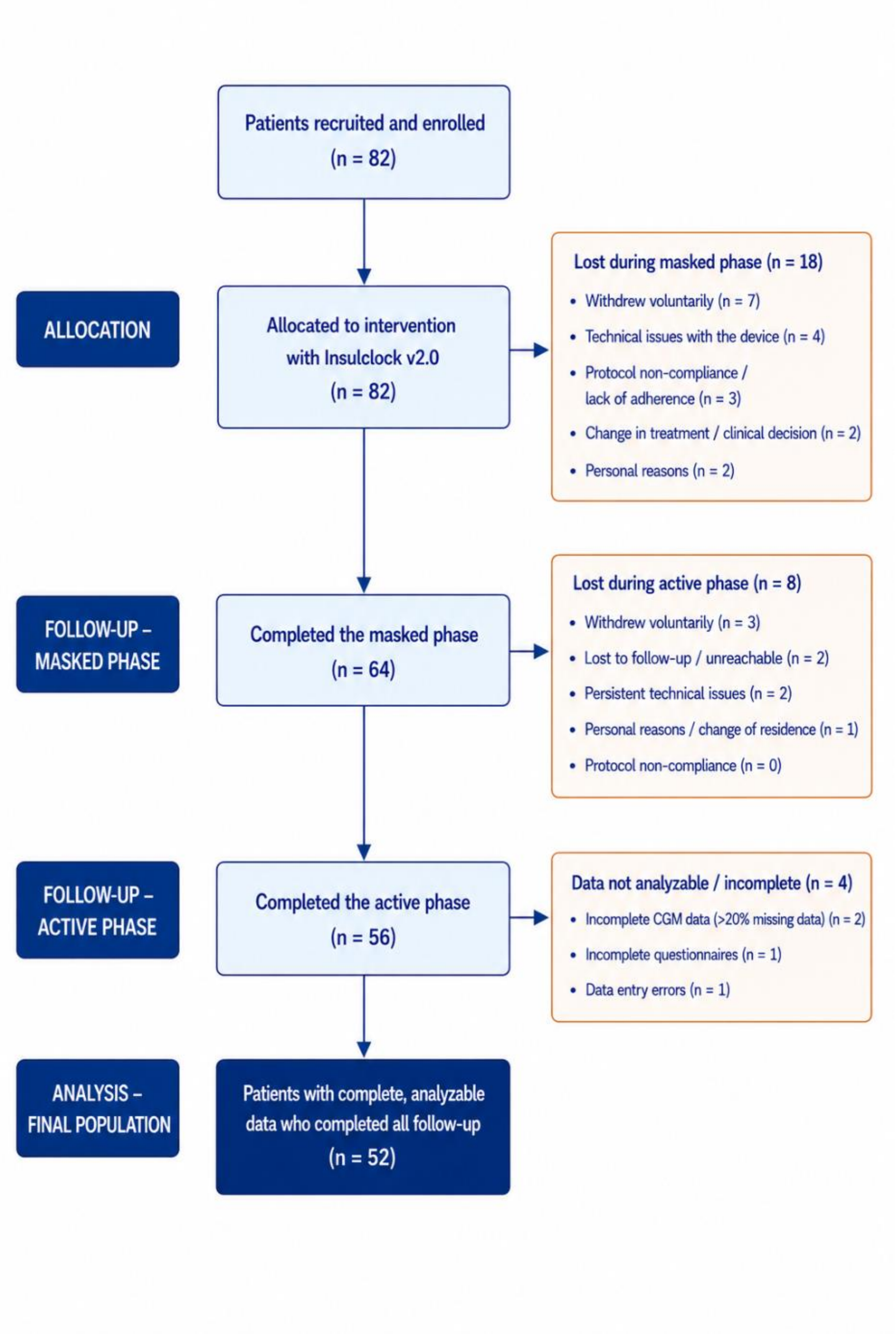

Supplementary Figure 2. Scatter plots of TIR, TAR180 and TBR70 and insulin injection time in relation to meal intake.

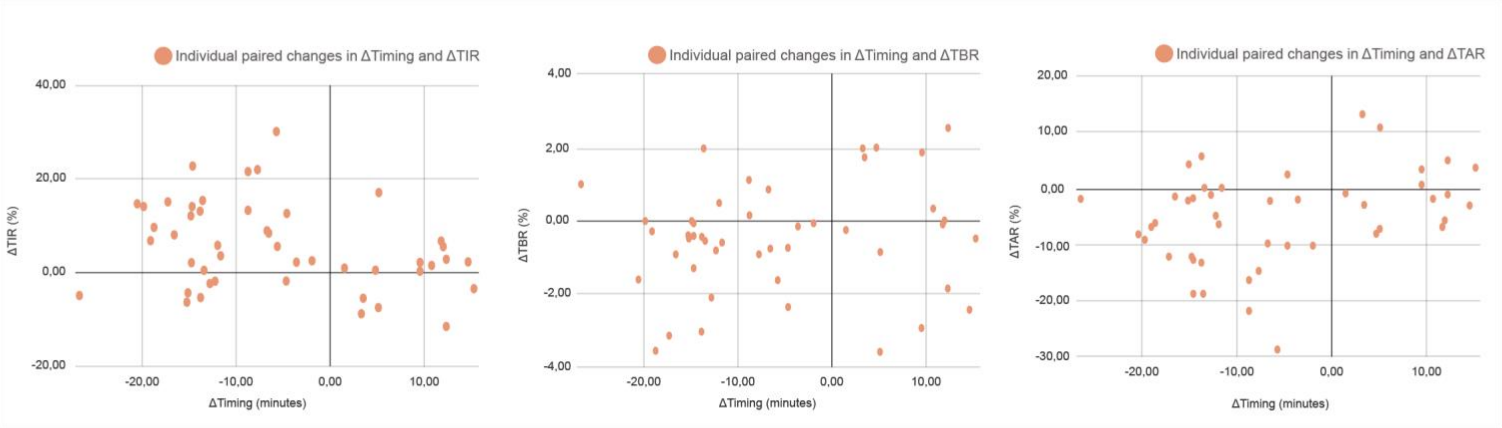

Supplementary Figure 3. Violin plots showing the distribution of insulin doses and injection among subjects by study phase and rapid insulin type use.

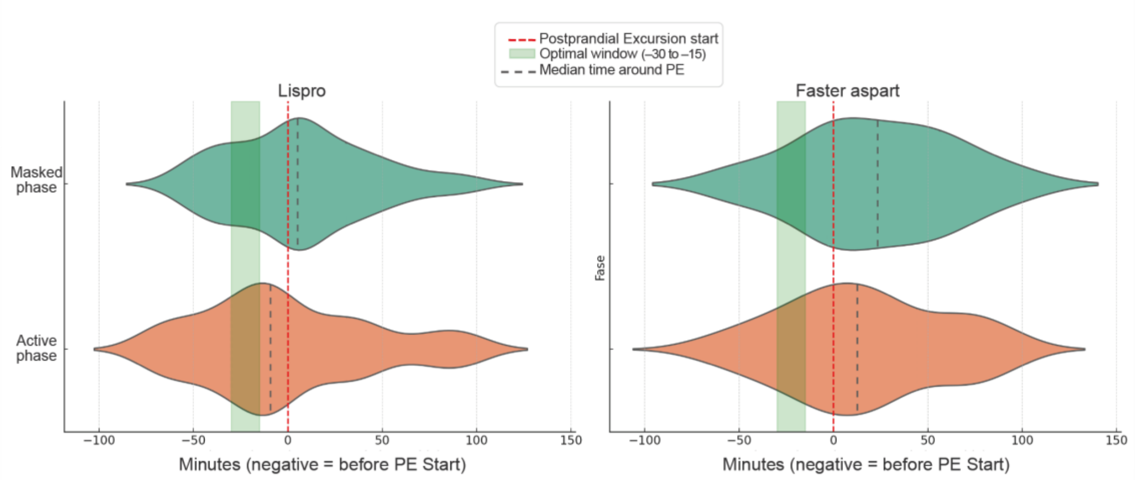

Supplementary Figure 4. ITSQ items change between masked and active phases.

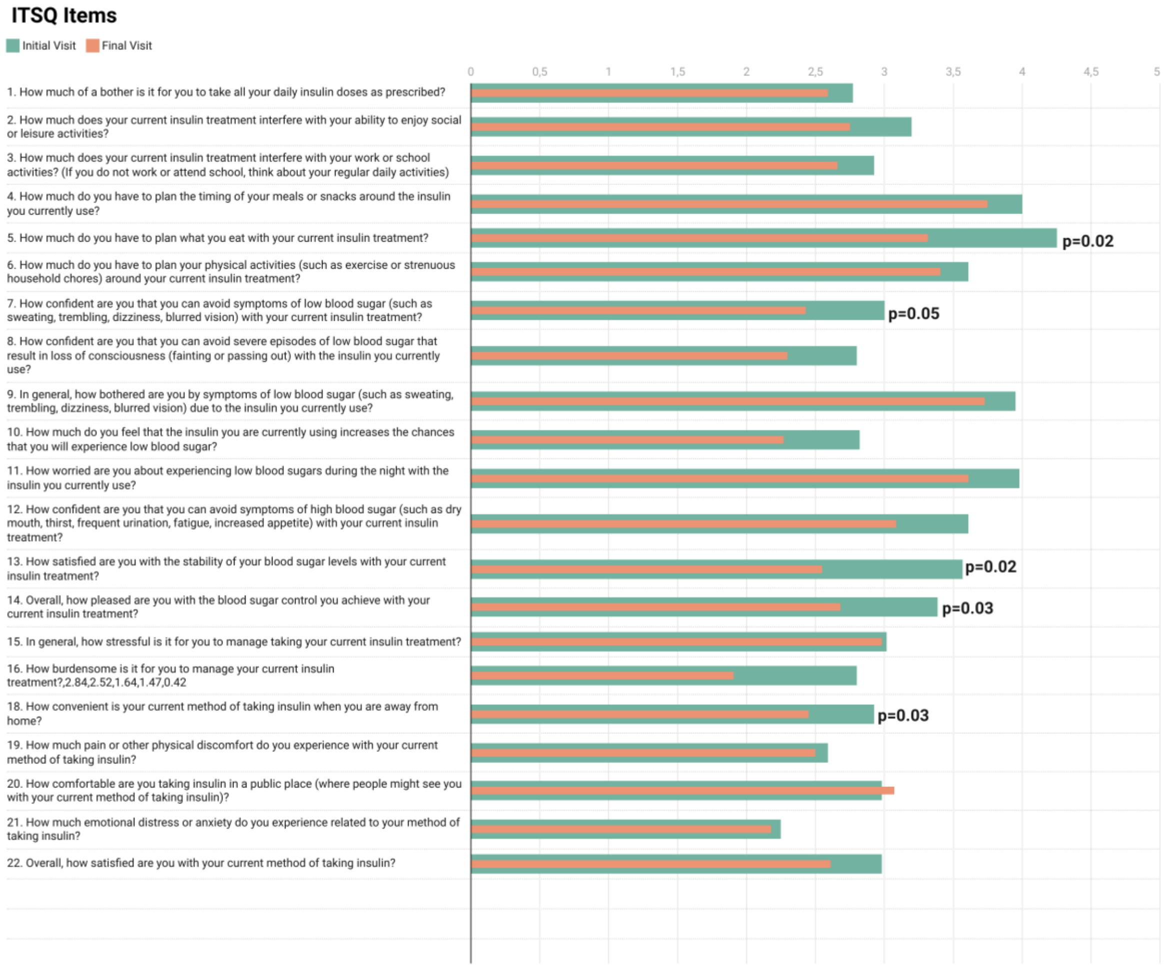

Supplementary Figure 5. Patient-reported satisfaction with diabetes treatment regimens (PRSD) items change between masked and active phases.

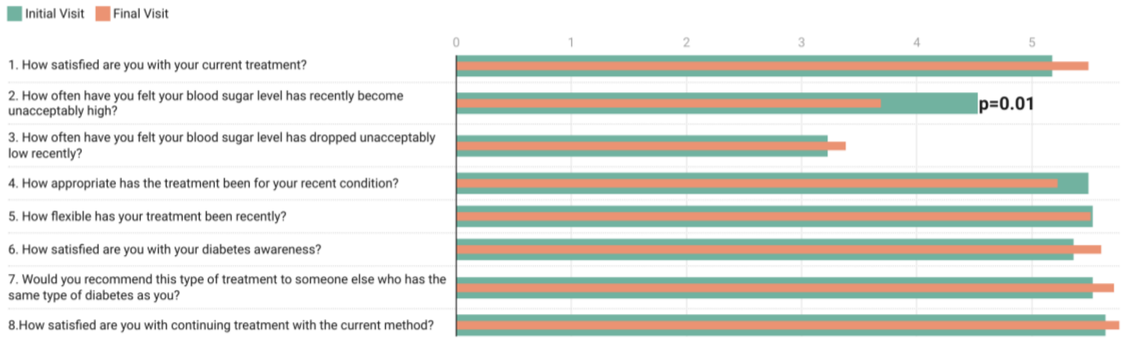

Supplement: Supplementary file 1 [file DataSheet1.pdf]
